# Diabetes Mellitus

**Published:** 1900-11

**Authors:** R. Alexander Bate

**Affiliations:** Assistant to the Principles and Practice of Medicine and Clinical Medicine, Hospital College of Medicine, Louisville, Ky.


ATLANTA

### Journal-Record of Medicine.

Successor to Atlanta Medical and Surgical Journal, Established 1855, and Southern Medical Record, Established 1870.

VOL. II. NOVEMBER, 1900. NO. 8.

EDITORS { BERNARD WOLFF, M.D., DUNBAR ROY, A.B., M.D.

M. B. HUTCHINS, M.D., BUSINESS MANAGER.

Published Monthly. Office No. 64 Marietta Street, Opposite Post-office.

### *ORIGINAL COMMUNICATIONS.*

### DIABETES MELLITUS.*

BY R. ALEXANDER BATE, A.B., M.D.,

Assistant to the Principles and Practice of Medicine and Clinical Medicine, Hospital College of Medicine, Louisville, Ky.

Diabetes mellitus is a disease of malassimilation, in which deficient oxidation either directly or indirectly permits the accumulation of sugar in the blood.

Sugar is normally produced in the system by the action of the saliva, the pancreatic and intestinal juices and by the liver. It is put into the circulation to be consumed by oxidation.

If sugar accumulate in the blood in a proportion greater than three parts per thousand, it is excreted in the urine.

Diabetes may result either from an overproduction of sugar in

*Read before the Tri-State Medical Society of Georgia, Alabama and Tennessee. October 11, 12 and 13th, Chattanooga, Tenn.

the system or from a diminished power of oxygenation. Either more sugar is produced than is acted upon by a normal amount of oxygen; or the amount of oxygen is too greatly diminished to convert a normal amount of sugar.

Consequently such things as affect either the production of sugar in the system or its oxygenation may be mentioned as predisposing causes of diabetes mellitus. Excessive quantities of saccharine, starchy or nitrogenous food tend toward an overproduction of sugar in the system.

Sedentary occupations, diseases of the lungs, faulty hygiene or environment, together with hereditary causes, lessen oxidation.

Lyman says the influence of heredity is as apparent in diabetes as in the other disorders of nutrition belonging to the arthritic diathesis. It has been observed in as many as three consecutive generations.

Hebrews show a radical predisposition. Men are more frequently affected than women. Children are rarely affected. The disease occurs most frequently between the ages of thirty and sixty.

Obesity and diabetes frequently occur in the same individual. Osler reports cases in which the diseases were associated for three consecutive generations.

Gout and rheumatism have also been observed in the subjects of diabetes. In osteomalacia it is believed improved hygiene oxidizes the lactic acid one step farther (into sugar), and the osteomalacia of the tenement house may become diabetes in a sanitary institution.

Among the actual causes of saccharine diabetes may be mentioned the arthritic diathesis, mental strain and injury to the spinal cord or brain and particularly diseases involving the region of the floor of the fourth ventricle.

Excessive activity in the glycogenic function of the liver or in any of the primary assimilative forces, perhaps, rarely overloads the blood with sugar.

Hausemann found lesions of the pancreas in about fifty per cent. of cases. The removal of the pancreas in the dog has been repeatedly followed by the appearance of sugar in the urine.

Harris (in the *American Year Book of Medicine* for 1900)

reports two cases of diabetes following diseases of the salivary glands. The removal of the salivary glands in the dog also caused sugar to appear in the urine.

Physiologists have demonstrated that puncture of the floor of the fourth ventricle causes increased circulation in all the abdominal viscera.

This apparently shows that irritation to the central nervous system does not cause an increase in the glycogenic function of the liver, but simply causes more blood to flow over the sugar-forming areas.

In the arthritic diathesis perhaps two factors tend toward the production of diabetes. First: The power of oxygenation is greatly diminished. Second: The presence of uric acid in the blood causes high arterial tension, which Haig has shown produces venous congestion, especially of the liver. So that a condition results similar to the one produced by cerebral irritation. Only the puncture of the floor of the fourth ventricle causes a greater quantity of arterial blood to flow through the liver, and the high arterial tension causes an increased quantity of venous blood to be held in contact with the sugar-forming areas.

The characteristic lesion of diabetes is the presence of sugar in the blood, in varying proportions up to nine or ten parts in a thousand, and, secondarily, in all the organs, secretions and excretions of the body. (Loomis.)

Glycogen, acetone, kreatin, and fat in excess are present in the various fluids. The blood is usually loaded with uric acid; parenchymatous degeneration occurs in the viscera from the altered condition of the blood. The liver is found enlarged and hyperemic, with signs of fatty degeneration. The lungs are frequently tuberculous, with localized catarrhal pneumonia, pleurisy, or gangrene. The heart is soft, flabby, and sometimes hypertrophied. The spleen is hyperemic, hypertrophied and firm. The kidneys also become hyperemic, and are the seat of parenchymatous changes. Softening, cirrhosis, tumors and cysts have been found in the brain, and rarely multiple neuritis of the cranial nerves exists. The pancreas may be atrophied and sometimes the pancreatic ducts are occluded by calculi. Dilatation of the stomach is common.

Emaciation takes place early and is marked. The proliferation of pyogenic microphytes in the saccharine fluids causes balanitis, vulvitis, and various cutaneous disorders, among which are certain forms of lichen. The skin becomes dry, and is the seat of furuncles, carbuncles, bed-sores, and gangrene. Cataract, retinal hemorrhage and degeneration of the optic nerve and retina are the lesions that occur within the eyeball.

The symptoms of diabetes are due to the dehydrating action of sugar upon the various tissues of the body, to the high arterial tension of uric-acidemia and to autointoxication.

The symptoms first attracting the attention of the patient—the increased amount of urine voided, the thirst, dryness of mouth, throat and skin, together with the itching and loss of flesh—are perhaps due to the dehydrating action of sugar.

The excess of acids and products of incomplete metabolism, the neurotic disturbances and the subnormal temperature and pulse are, perhaps, manifestations of uric-acidemia.

General autointoxication characterized by the presence of oxybutyric acid in the system causes diabetic coma.

Diabetes mellitus cannot be mistaken for any other disease. Repeated urinalyses eliminate simple glycosuria and render a diagnosis positive.

The prognosis concerning saccharine diabetes is more favorable than formerly believed. As yet few cures are recorded; but the consensus of opinion points to a general amelioration of symptoms, and in many instances life is greatly prolonged by modern therapy.

The younger the subject the more unfavorable the prognosis.

The transmitted form of diabetes is not only more severe, but there is an inherited inability to resist nutritional disorders.

This marks the contrast between constitutional diseases due to faulty metabolism and those of microbic origin.

The progeny of those affected with nutritional disorders become progressively more puny and more susceptible to metabolic disturbances, while the progeny of those continually exposed to microbic invasion frequently manifest immunity.

The treatment of diabetes mellitus demands attention to the primary cause, and the use of such dietetic, hygienic and therapeutic measures as promote oxidation and assimilation, and overcome arterial tension.

When possible any irritant to the central nervous system should be removed, and pulmonary or other disease interfering with oxidation should be arrested, and dietetic and hygienic errors retarding metabolism and oxidation should be corrected.

The dietetic treatment consists in the use of such articles of food as contain most oxygen and are free from sugar and uric-acid forming material.

Saccharine and starchy food, red meats, coffee, tea, strawberries, bananas, chestnuts, and the rest of the alloxan group should be avoided. White meats, lentils, eggs, milk, breads well browned and free from yeast, together with fruits, and nuts generally, may be permitted.

Such hygienic measures should be pursued as best promote oxidation. A life in the open air and sunshine should be selected in a warm dry climate.

Ocean voyages, mountain climbing, bathing and massage are recommended on account of promoting oxidation. The therapeutic measures embrace not only the control of the excretion of sugar and its dehydrating action, but treatment of the arthritic diathesis and attention to the special symptoms arising in individual cases.

The uric-acidemia, which causes high arterial tension and venous engorgement, must be overcome by eliminating uric acid from the system.

The salicylates, the benzoates, salts of lithia, piperazin and other uric acid solutions are serviceable. Haig reports marked benefit from the use of the salicylates. All the drugs used at present to lessen the excretion of sugar are drugs that also clear the blood of uric acid. The mistake previously made was in stopping at this result and not adding treatment to remove the uric acid from the system.

The drugs lessening the excretion of sugar are opium and its alkaloids, quinine sulphate injected into the veins, certain salts of arsenic, and jambul.

Potassium permanganate and oxygenated waters act favorably by increasing oxidation.

Pancreatic extract has proven beneficial in those cases where the pancreas was involved.

Extract of liver has been used, but may be contraindicated in cases due to an overproduction of sugar.

Suprarenal extract is indicated, as in other disturbances of the arthritic diathesis on account of its tonic action upon the cardio-vascular system. Benzosol and creosote are employed to control fermentative changes.

The presence of oxybutyric acid in the system is best controlled by large quantities of alkalies, which may be administered intravenously or otherwise.

The points it has been desired to emphasize are:

That modern opinion seems to regard diabetes as due to a deficient power of oxygenation dependent upon the arthritic diathesis.

That arthritism not only lessens oxidation, but causes more sugar to be taken up by the blood by increasing the quantity of blood brought into contact with the sugar-forming areas.

That antilithemic diet and treatment are not only rational but afford the best results; and that the alkalies are most useful to control the diabetic coma.

I have previously reported cases of glycosuria occurring in lithemic individuals in which cures were effected by antilithemic remedies.

The cases of diabetes mellitus thus treated have appeared to me to excrete less sugar, to miss polyuria, furunculosis, thirst, emaciation, pruritus, and the severer symptoms generally, and life has seemed to be prolonged.

